# RiPa-Net: Recognition of Rice Paddy Diseases with Duo-Layers of CNNs Fostered by Feature Transformation and Selection

**DOI:** 10.3390/biomimetics8050417

**Published:** 2023-09-07

**Authors:** Omneya Attallah

**Affiliations:** Department of Electronics and Communications Engineering, College of Engineering and Technology, Arab Academy for Science, Technology and Maritime Transport, Alexandria 1029, Egypt; o.attallah@aast.edu

**Keywords:** paddy disease, deep learning, convolutional neural networks, rice disease recognition, smart agriculture

## Abstract

Rice paddy diseases significantly reduce the quantity and quality of crops, so it is essential to recognize them quickly and accurately for prevention and control. Deep learning (DL)-based computer-assisted expert systems are encouraging approaches to solving this issue and dealing with the dearth of subject-matter specialists in this area. Nonetheless, a major generalization obstacle is posed by the existence of small discrepancies between various classes of paddy diseases. Numerous studies have used features taken from a single deep layer of an individual complex DL construction with many deep layers and parameters. All of them have relied on spatial knowledge only to learn their recognition models trained with a large number of features. This study suggests a pipeline called “RiPa-Net” based on three lightweight CNNs that can identify and categorize nine paddy diseases as well as healthy paddy. The suggested pipeline gathers features from two different layers of each of the CNNs. Moreover, the suggested method additionally applies the dual-tree complex wavelet transform (DTCWT) to the deep features of the first layer to obtain spectral–temporal information. Additionally, it incorporates the deep features of the first layer of the three CNNs using principal component analysis (PCA) and discrete cosine transform (DCT) transformation methods, which reduce the dimension of the first layer features. The second layer’s spatial deep features are then combined with these fused time-frequency deep features. After that, a feature selection process is introduced to reduce the size of the feature vector and choose only those features that have a significant impact on the recognition process, thereby further reducing recognition complexity. According to the results, combining deep features from two layers of different lightweight CNNs can improve recognition accuracy. Performance also improves as a result of the acquired spatial–spectral–temporal information used to learn models. Using 300 features, the cubic support vector machine (SVM) achieves an outstanding accuracy of 97.5%. The competitive ability of the suggested pipeline is confirmed by a comparison of the experimental results with findings from previously conducted research on the recognition of paddy diseases.

## 1. Introduction

More than 50% of the global population relies on rice as their primary source of nutrition [1,2]. It makes up thirty-six percent of all essential foods consumed worldwide, and the need for it is expected to increase in the coming years [1]. Paddy agriculture is impacted by numerous diseases. Both farmers and agricultural professionals face challenges in the timely identification of these paddy conditions [3]. Agriculturalists have historically used manual methods relying on their knowledge and sight to determine paddy illnesses, but these methods are incredibly ineffective, laborious, and susceptible to mistakes [4]. Because of a broad spectrum of signs that are the same, paddy diseases can sometimes be difficult to precisely recognize despite the efforts of skilled farmers as well as agricultural specialists [5]. Therefore, a straightforward, efficient, and automated tool or technological advance is required to alleviate these important concerns for agriculturalists.

A paradigm shift is required in agriculture that utilizes cutting-edge concepts and technologies to address the problems mentioned above in a sustainable manner at affordable prices without harming the ecosystem. To this end, this study is inspired by the success of artificial intelligence (AI) methods, such as deep learning (DL) and machine learning, in multiple areas involving medicine [6,7,8,9,10], healthcare [11,12,13,14], machine fault diagnosis [15,16], and industry [17,18]. These techniques have been adopted to revolutionize agriculture. DL has become a powerful method to solve a variety of computer vision problems. Furthermore, convolutional neural networks (CNNs), an example of DL models, have demonstrated promising efficiency for the detection of crop diseases. Throughout the past couple of decades, a number of DL-driven disease detection approaches have recently been established [19,20,21]. The CNN models are potentially challenging to put into operation for real-time and live plant disease identification and evaluation employing light computational tools. Regular CNN structures with large deep layers require a lot of computational capability and possess a big storage footprint. If lightweight CNN structures with a smaller amount of layers and parameters are capable of identifying illnesses with an equivalent level of accuracy, they may be an acceptable substitute [22].

For the aforementioned reason, this study aims to propose a pipeline entitled “RiPa-Net” based on numerous compact CNNs with fewer layers and fewer parameters that are capable of detecting and classifying rice diseases in agricultural paddy areas. The proposed pipeline acquires features from two distinct deep layers of each compact CNN instead of obtaining features from a sole layer like in other studies on the detection of plant disease [23,24,25,26,27,28]. Furthermore, in order to attain a spectral–temporal illustration of the input data instead of relying on spatial information provided by CNN, the proposed pipeline employs dual-tree complex wavelet transform (DTCWT) on deep features obtained from the first layer, which is also employed to reduce the complexity of the classification by diminishing the size of the deep features. The proposed pipeline uses transformation methods including principal component analysis (PCA) and discrete wavelet transform (DCT) to fuse deep features obtained from the first layer of the three CNNs, which also lower feature size. The proposed pipeline then concatenates the spectral–temporal deep features of the first layer with those of the second deep layer. Next, it employs a feature selection procedure to reduce the dimension of the concatenated features, which correspondingly reduces the complexity of the classification process.

The following highlights the originality and contribution of the study:

- Introducing an efficient and reliable pipeline to detect and classify nine paddy diseases based on three lightweight CNNs instead of a single DL model.
- Acquiring deep features from dual distinct deep layers of each CNN rather than obtaining deep features from one layer.
- Relying on spatial–spectral–temporal deep features as a replacement for only spatial features by adopting DTCWT to analyze and reduce deep features acquired from the first deep layer of each CNN and then concatenating it with deep features of the second layer.
- Employing PCA and DCT transformation methods that merge deep features of the three CNNs and reduce the dimension of deep features, thus reducing the training complexity of the recognition models.
- Blending deep features of the three CNNs to perform classification rather than depending on the deep features of a single CNN.
- Presenting a feature selection process to choose only the persuasive features and ignore unnecessary features, thus decreasing the classification complexity.

The remaining sections of the paper are arranged as follows: Section 2 presents recent previous work on paddy disease recognition. Next, Section 3 describes the methods and materials employed in this study, including the suggested pipeline. After that, Section 4 demonstrates the dataset description, parameter fine-tuning, and performance metrics utilized to access the suggested pipeline. Afterward, Section 5 illustrates the results of the suggested pipeline. Subsequently, Section 6 discusses the main findings of the suggested pipeline. Finally, the last section concludes the paper.

## 2. Previous Work on Paddy Disease Recognition

CNNs have proven their effectiveness in several image analysis applications [29,30,31,32], which is why they are an excellent choice to help examine paddy diseases in smart agriculture [33]. An overview of the related work dependent on various CNN variations is provided in this part of the paper. For example, the study [24] compared the performance of 13 CNN models of different architectures in the detection of rice diseases. The deep features extracted from these models were used independently to train a support vector machine classifier (SVM). The features of a Residual Network having 50 deep layers (ResNet-50) achieved the highest accuracy of 98.38%. Likewise, the SVM classifier was fed with deep features obtained from Alex Network (AlexNet) CNN to classify rice diseases reaching an accuracy of 96.8%. Additionally, research articles [34,35] acquired deep features using a custom CNN to detect and classify growing diseases, achieving accuracies of 92.23% and 99.2%, respectively. The authors of the study [36] modified the Re-parameterization Visual Geometry Group (RepVGG) CNN model by adding an efficient channel attention mechanism to improve the performance in detecting rice diseases accomplishing an accuracy of 97.06%.

In addition, the study [37] compared the performance of a custom CNN with traditional machine learning algorithms based on handcrafted feature extraction and segmentation approaches. The results showed that CNN outperformed traditional machine learning methods, reaching 87.5% accuracy. On the contrary, the research article [38] detected key points from paddy images and then extracted deep hypercolumn features from the VGG CNN model. Finally, classification was performed using artificial neural network (ANN), SVM, and random forest (RF) classifiers, where RF attained a maximum accuracy of 93%. The researchers of [39] employed GoogleNet and VGG-16 separately to detect and classify several rice diseases, achieving accuracies of 92.24% and 91.28%, respectively. On the other hand, the study [40] proposed a new deep learning framework where features were extracted using a customized CNN and classified using a long short-term memory (LSTM) DL model, achieving an accuracy of 97.0%. The study [41] acquired deep features of VGG-16 and fed an SVM classifier to classify rice diseases with an accuracy of 97.31%. On the other hand, the study [42] optimized a CNN using a mutant particle swarm optimization approach to detect rice disease accomplishing 93.35% accuracy.

It can be noted that most of the studies mentioned above depend on only spatial information to detect and classify paddy disease, while employing spectral–temporal demonstration could enhance the classification process. Additionally, most of the existing studies employed CNNs of large dimensions and parameters, whereas using compact CNNs with a minor amount of layers and parameters while achieving high accuracy is better as it reduces the complexity of classification [22]. Furthermore, most current models for paddy diseases relied on a single DL model; however, depending on an ensemble of DL models may boost classification results [9,43]. Also, the present studies obtained deep features from a single layer of a DL model, nevertheless acquiring deep features from more than one layer could enhance performance [44]. Moreover, most of them did not employ feature selection to decrease feature space size and lessen the complexity. Furthermore, many studies classified a few paddy diseases due to the lack of a public dataset that contains numerous paddy diseases. To address the aforementioned shortcomings, this study proposes RiPa-Net, which is a pipeline based on three lightweight CNNs to detect and recognize nine paddy diseases as well as normal paddy. Opposing current studies, the proposed pipeline acquires features from two different layers of each of the CNNs. Rather than relying solely on spatial knowledge to classify paddy diseases, the proposed pipeline applies the dual-tree complex wavelet transform (DTCWT) to deep features of the first layer to obtain spectral–temporal information. DTCWT is also used to lessen the dimension of deep features extracted from the first layer and lower the complexity of recognition. Instead of employing the deep features of each CNN independently to perform recognition, the proposed pipeline combines the deep features of the three lightweight CNNs. It utilizes PCA and DCT transformation methods to combine deep features of the first layer of the three CNNs, which also reduces the dimension of features obtained from the first layer. It then concatenates these fused time-frequency deep features after the PCA or DCT with the spatial deep features of the second layer. Afterward, it introduces a feature selection procedure to diminish the feature vector size and select only those influential features that affect the classification process, which accordingly reduces the classification complexity further. 

## 3. Materials and Methods

### 3.1. Transformation and Reduction Methods

#### 3.1.1. Dual-Tree Complex Wavelet Transform

In a number of fields, the discrete wavelet transform (DWT) has demonstrated effective analyzing ability. With DWT, minor changes in input data (*x*) may end up in considerable variations in the proportion of energy of coefficients associated with wavelets, where this problem is known as shift variance [45]. To solve the issue, DTCWT was proposed in [46], which analyses the input data by employing a pair of distinct DWT transformations [47,48]. The DTCWT method consists of two real DWTs: the first DWT provides the real coefficients of the transformation, and the second DWT presents the imaginary portion. Sub-bands of the higher frequency DWT are required to be used in the DTCWT filters to ensure that the real component of the DTCWT can be explained, whereas lower DWT frequency sub-bands demonstrate the fictitious portion of the DTCWT. The transformation is increased by a weight of 2, and the shift’s invariant is maintained [45]. Take into account that for the real part, {*l_o_*(*x*), *h_o_*(*x*)} stands in for the low-pass–high-pass filter couple, while for the imaginary part, {*l*_1_(*x*), *h*_1_(*x*)} presents the same. The two real wavelets that correspond to the respective values of the two real wavelet transformations are denoted by the letters *Ψ*_l_(t) and *Ψ*_h_(t). Filters are constructed so that roughly speaking, *Ψ*_l_(t) is the Hilbert transform of *Ψ*_h_(t) represented as *Ψ*_l_(t) ≈ *H*(*Ψ*_h_(t)). It is intriguing that since the filters are real, the DTCWT can be implemented without the need for complex calculations.

#### 3.1.2. Principal Component Analysis

Massive datasets are increasingly prevalent than ever and are often challenging to comprehend. A method to decrease the size associated with these databases, improving comprehension while minimizing the loss of information, is PCA. The eigenvector is an analytical approach that PCA employs to determine the direction of attributes. The basic idea behind PCA is to convert a *j*-dimensional feature dimensionality into a lower *i* dimension called principal components, where *i* < *j*. In order to compute the principal components, a covariance matrix is initially determined along with the eigenvectors. Afterward, the eigenvector with the highest eigenvalue is chosen as the principal component since it demonstrates the strongest correlation among the data features. The principal component(s) with the highest eigenvalues are chosen, and the eigenvalues with the lowest values are ignored. It accomplishes this by producing novel, non-correlated variables that maximize variability one after the other [49,50]. 

#### 3.1.3. Discrete Cosine Transform

According to [51], DCT is a form of linear transformation approach commonly used in the signal processing area for compressing and compacting the energy of a signal. DCT removes disruptive and inefficient values while storing an enormous quantity of data within the low-frequency portion of the input data [52]. It enables the breakdown of the input data into its fundamental frequency elements. The input data are expressed in the DCT as a linear set of weighted basic functions connected to its spectral elements. Due to the fact that it contains different values, the low-frequency portion is more useful [53]. After analyzing the input data using DCT, a DCT coefficients matrix is generated. Only some of the coefficients are kept and the rest of them are ignored. The feature reduction procedure includes a crucial step called the choice of the DCT coefficients. Typically, traditional techniques, like zigzag, are employed to choose among the DCT coefficients [54].

### 3.2. Suggested RiPa-Net Pipeline

**Figure 1:**
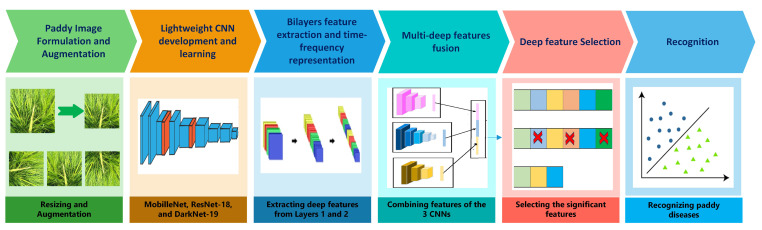
Phases of the proposed RiPa-Net pipeline.

The suggested RiPa-Net pipeline is composed of six phases. These phases are initially the paddy image formulation and augmentation, in which RGB images of the ten categories of paddy disease categories are processed. Additionally, the dimensions of these images are changed to fit the input size of the lightweight CNNs, and then augmentation methods are applied to them. Next, there is the lightweight CNN development and learning phase, where three compact CNNs are created involving the Mobile Network (MobileNet), ResNet-18, and the Dark Network of 19 deep layers (DarkNet-19) and learned with these augmented images. Afterward comes the bilayer feature extraction and time-frequency representation phase. At this stage, for every CNN, deep features are acquired from two distinct layers: layers 1 and 2. Layer 1 produces features of large dimensions; thus, DTCWT is employed to reduce the dimensionality and represent data in the spectral–temporal domain instead of depending on spatial information alone. Subsequently, there is the multi-deep feature fusion phase, where the deep features of layer 1 of the three CNNs are merged using two reduction approaches, including PCA and DCT. Also, layer 2 features are concatenated and integrated with layer 1 spatial–spectral–temporal features. After that is the deep feature selection, and in this stage, a feature selection approach is applied to choose those features that are more important to diagnostic performance. Finally, there is the recognition phase, in which three support vector machine classifiers are employed to perform the recognition step. Figure 1 visualizes the phases of the proposed pipeline.

The pseudocode of the suggested pipeline is described in Algorithm 1:
**Algorithm 1.** The steps of the proposed RiPa-Net pipeline.**Input:** Paddy RGB Images **Output:** Recognized paddy diseases1. **Begin RiPa-Net**:2. Resize all images to fit the input size of the CNNs: 224 × 224 × 3 for MobileNet and ResNet-18 and 256 × 256 × 3 for DarkNet-19.3. Augment images to avoid overfitting and boost CNNs performance: rotation, flipping, shearing, and scaling.4. Create lightweight CNN models including MobileNet, ResNet-18, and DarkNet-195. Set some CNN hyperparameters: learning rate (0.0001), mini-batch (10), epochs (20), and validation frequency (778).6. **Start**: CNN learning process:7. After the learning process is finished, End of the learning process.8. **For each CNN**:9. Extract deep features from layer 1 and layer 2.10. Apply DTCWT to layer 1 features to obtain spatial–spectral–temporal deep features.11. **End For**12. Fuse Layer 1 deep features of the three CNNs: using PCA and DCT to fuse and reduce feature space dimensionality.13. Concatenate deep features of the previous step with deep features of layer 2 of the three CNNs.14. Apply mRMR feature selection to select the most significant features.15. Construct classifiers: linear SVM, quadratic SVM, and cubic SVM.16. Test classifiers: recognize paddy disease using the testing set (use 5-fold cross-validation).17. **End RiPa-Net**

#### 3.2.1. Paddy Image Formulation and Augmentation

In the beginning, the dimensionality of the photos from the Paddy Doctor dataset is altered to suit the dimension of the CNN input layers. These aspects are equal to 224 × 224 × 3 for MobileNet and ResNet-18 and 256 × 256 × 3 for DarkNet-19. These data are then split into a 70–30% ratio for training and testing portions. The training images are then augmented to expand the number of photographs. This augmentation step is necessary to enhance the learning experience of the CNNs and stop overfitting. Several augmentation methods are applied to the training split. These augmentation methods and their values are presented in Table 1. All the augmentation techniques are applied to training images only that are used to train the CNNs and not for testing images. This is performed to ensure that a replica of the image is not included in the training and testing and to avoid over-optimistic results. It is worth mentioning that the augmentation utilized in this study is the MATLAB online augmentation technique. This means that this technique incorporates augmentation while training the model. This indicates that the model is supplied with randomly chosen batches of the original dataset during each epoch, and the transformations are then carried out online. Additionally, the photos given to the model for each epoch vary according to the transformations used. In other words, the augmentation procedure in MATLAB is integrated into the data store, and the results of the augmentation are not shown or stored in memory. This happens as MATLAB does not perform the typical increase in photos in memory. The MATLAB group employed the concept of data augmentation taking into account a memory-constrained computer. The dataset used in this study is described later in Section 4.1.

#### 3.2.2. Lightweight CNN Development and Learning

Three lightweight CNNs are developed using transfer learning (TL), such as Mobile-Net, ResNet-18, and DarkNet-19. The TL methodology is used for transferring the information that is learned during the development phase of CNNs learned with massive datasets to address a similar classification issue via a database that contains relatively few photos to learn the model. TL aids in avoiding the convergence process and problems associated with overfitting. This procedure typically improves the accuracy of recognition [55]. DL models that employ TL are called pre-trained models. TL is adopted in this study to change the output layer of CNNs to 10 similar to the number of dataset categories. Augmented images formed in the previous stage are then utilized to learn the pre-trained CNNs after adjusting some CNNs’ hyperparameters, including mini-batch, learning rate, epochs, and validation frequency. More details regarding the hyperparameters will be discussed later in Section 4.2.

#### 3.2.3. Bilayers Feature Extraction and Time-Frequency Representation

After ending the learning progression of the three CNNs, TL is again used to extract deep features from two different deep layers of each CNN. This part aims to examine whether extracting deep features from more than one layer of a CNN is superior to obtaining deep features from a single deep layer of a CNN. In addition, this section examines which of the deep layers has a greater impact on classification accuracy. The last two deeper layers are employed in this step. This is because deeper layers obtain more detailed and substantial information in contrast to primary layers which learn elementary representations from the input data [56]. Layer 1 signifies the final pooling layer of MobileNet and ResNet-18, while it is the last convolution layer of DarkNet-19, whereas layer 2 represents the ultimate fully connected (FC) layer of MobileNet and ResNet-18 and the last pooling layer for DarkNet-19. Features acquired from these layers demonstrate only spatial information of the input; however, as mentioned earlier, obtaining a spatial–spectral–temporal representation of the input is superior to using only spatial demonstration and could enhance performance. Table 2 displays the size of the feature vector obtained from each layer of the three CNNs. The dimension of the features of layer 2 is greater than that of layer 1, as shown in Table 2. For this reason, DTCWT is employed to reveal the spatial–spectral–temporal representation of the layer 1 features. Furthermore, it is used to compress their size. Note that two levels of decomposition were employed from DTCWT, where the lowpass coefficients of the second level are chosen as the reduced spatial–spectral–temporal features.

#### 3.2.4. Multi-Deep Features Fusion

In this phase, rather than relying on the deep features of a single CNN architecture, the deep features of each deep layer of the three CNNs are incorporated separately. This integration process combines all the privileges of the three CNN structures, which usually improves performance. The fusion procedure for layer 1 features is attained using two transformation algorithms, which are PCA and DCT. These two methods are compared to demonstrate that fusion can enhance performance. These approaches not only merge features, but also diminish their size. To demonstrate how changing the number of fused features affects performance, an ablation study is conducted. On the other hand, the deep features of layer 2 for the three CNNs are concatenated, as they have low dimensions and do not require a reduction step. 

#### 3.2.5. Deep Feature Selection

Next comes the deep feature selection step, where the aim of this phase is to diminish the dimensionality of the feature space and reduce training time duration and complexity of the training time. In this phase, the features of each deep layer of three CNNs integrated with the previous step are then concatenated, and a feature selection process is applied to them to choose a reduced set of features that impact the recognition performance. The term “feature selection” describes a group of computational methodologies, the objective of which is to choose the most relevant attributes from the initial feature set [57]. Feature selection is a useful technique for handling data that is highly multi-dimensional because it may decrease feature dimensionality and redundancy and help with problems like the overfitting of models in subsequent analysis. This is unlike dimension reduction techniques, such as PCA, which integrates and converts the dataset’s original variables to produce a smaller variable size. Feature selection techniques only determine and choose features that meet pre-established criteria or optimize particular computational techniques.

In the proposed pipeline, a maximum relevance minimum redundancy (mRMR) feature selection approach is used. The mRMR is a feature selection method that chooses attributes that are little in correlation with one another and have a significant association with the output label. For variable selection, the Pearson correlation coefficient may be utilized to determine the relationship among variables (redundancy), and the F-statistic may be applied to determine the association with the output label (relevance). The objective function, which is an equation of significance and redundant status, is then maximized by selecting variables iteratively using a greedy algorithm [58]. Mutual information is the objective function employed in this step.

#### 3.2.6. Recognition

In order to recognize the different paddy diseases in the Paddy Doctor dataset, three SVM classifiers of different kernels are operated. These kernels are linear, cubic, and quadratic. SVM is a well-known robust classifier. It is regarded as being one of the most well-known techniques for signal/image identification [59]. Because it employs a function called the kernel to translate the feature space into an alternative transformation that is capable of distinguishing among categories of data, it works effectively in massive dimension spaces and multi-class problems. As a result, it is frequently combined with the enormous size of deep learning attributes obtained from CNNs [60,61]. The recognition procedure is accomplished in three scenarios. In the first context, deep features obtained from layer 2 and layer 1 after applying DTCWT are used to feed the three SVMs. On the other hand, in scenario 2, the integrated features using the two fusion algorithms (PCA and DCT) are employed as inputs to the three SVMs. Later, in Scenario 3, the features selected using the mRMR feature selection approach are used to train the three SVMs.

## 4. Pipeline Setting 

### 4.1. Rice-Paddy Disease Dataset

**Figure 2:**
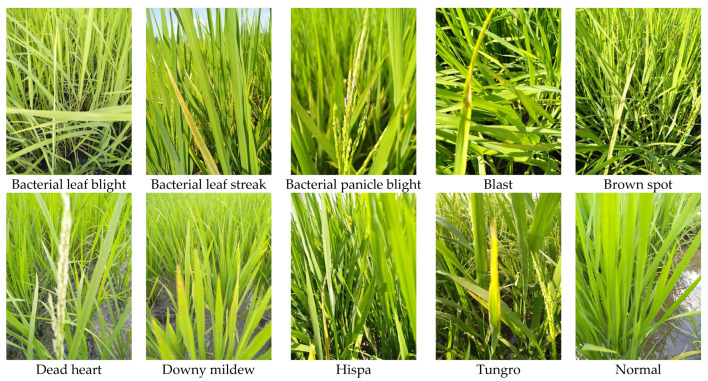
Examples of paddy diseases available in the Paddy Doctor dataset.

The dataset employed to validate the performance of the proposed model is called “Paddy Doctor” [62]. The dataset consists of nine paddy diseases along a tenth category consisting of normal images. The dataset includes RGB photographs taken in rice paddies in the Tirunelveli region of Tamilnadu, an Indian state. A total of 13,876 photos were taken; however, out of these images, only 10,407 were labeled with a specific category (normal or diseased with one of the paddy diseases), and thus, only these 10,407 labeled images were employed in this study. Each of these images has a resolution of 480 × 640. The rice diseases existing in the dataset, along with the total number of photos available for each disease, are indicated in Table 3. Figure 2 presents a representative example from every category of disease in the Paddy Doctor dataset. This figure represents a visual comprehension of the deviations existing throughout each class’s external appearance, such as various signs, phases, or harmed leaf portions. The observed fluctuations confirm the importance and difficulty of creating solid DL solutions that can precisely distinguish among diseases and generalize effectively to previously unknown deviations within a disease category.

### 4.2. Parameter Fine-Tuning

A few numbers of the CNNs hyperparameters are adjusted. These hyperparameters are the mini-batch, epoch size, learning rate, and validation frequency. These hyperparameters are adjusted to 10, 20, 0.0001, and 728 for the mini-batch, epoch size, learning rate, and validation frequency, respectively. Other parameters are kept with their default values. When a greater batch number is used, it was observed in [63] that the CNN model’s effectiveness, as measured by the network’s capacity to generalize, declines. Each of the learning and evaluation procedures eventually converges to razor-sharp minimizers when using larger batch lengths. Sharp minimal values have a negative impact on generalizability. Contrarily, the comparatively tiny number commonly coincides with soft minimizers and often accomplishes the highest generalization ability [64], so it is decided to limit it to a maximum of 10. In order to reach the smallest error, the learning rate advances while reflecting the factor of scaling at each epoch of learning. Rapid training times are made possible by high learning rates, but this results in an ultimate weight collection that is not ideal. In contrast, lower learning rates might actually enable the model to understand a slightly more optimal or even globally optimal collection of weights, leading to a comparatively prolonged training time. Additionally, high learning rates will lead to significant weight alterations, which will significantly alter the model performance across training runs. Efficiency varies as a result of weight variation. However, extremely low learning rates might never converge or might remain a less-than-ideal solution. As a consequence, the learning rate in the suggested pipeline is adjusted to 0.0001, which is neither low enough nor excessively big. The optimization technique implemented (SGDM) is stochastic gradient descent with momentum. Table 3 lists the rice diseases found in the dataset in addition to the overall amount of pictures provided for every illness.

To evaluate the effectiveness of the recognition models, a five-fold cross-validation and holdout test set is deployed. In five-fold cross-validation, the dataset is split into five equal subdivisions. Each time, the classifier is trained with four subdivisions and tested with the fifth sub-portion. This process is conducted five times; each time the training procedure is performed with four distinct subsets of data and tested with the remaining fifth subset. The testing accuracy is calculated each time; then, the average accuracy of the five testing folds is computed to evaluate the performance of the model. In the hold-out test set, 70% of the data is used for training and 30% is used for testing.

### 4.3. Assessment Measures

The effectiveness of pipeline recognition is measured using several assessment measures, including precision, F1-score, specificity, and the Mathew correlation coefficient (*MCC*). In order to calculate these measures, numerous indicators should be first determined, including true positive (*TP*), true negative (*TN*), false positive (*FP*), and false negative (*FN*). *TP* resembles the sum of correctly recognized positive labels, whereas *TN* signifies the total number of correctly identified negative labels. On the other hand, *FP* indicates negative labels incorrectly recognized as a positive class, while *FN* is the sum of positive label instances mistakenly classified as negative. The following formulas are adapted to calculate the assessment measures:

(1) $$Accuracy=\frac{TP+TN}{TN+FP+FN+TP}$$

(2) $$Sensitivity=\frac{TP}{TP+FN}$$

(3) $$Specificity=\frac{TN}{TN+FP}$$

(4) $$Precision=\frac{TP}{TP+FP}$$

(5) $$MCC=\frac{TP\times TN-FP\times FN}{\sqrt{\left(TP+FP)(TP+FN)(TN+FP)(TN+FN\right)}}$$

(6) $$F1\;Score=\frac{2\times TP}{\left(2\times TP\right)+FP+FN}$$

## 5. Results

This section demonstrates the results of three scenarios of the proposed RiPa-Net pipeline. In the initial scenario, the three SVMs are fed with deep features that are extracted from layer 1 or 2 after applying DTCWT. In contrast, the combined attributes created using each of the two integration methods (PCA and DCT) are used as inputs to the three SVMs in scenario 2. Later, in scenario 3, the three SVMs are trained using features chosen using the mRMR feature selection strategy.

### 5.1. Results of the First Scenario 

The target of this scenario is to investigate and compare the recognition performance of the trained SVM classifier with the deep features of two different layers. The results of the first scenario are discussed in this section. Table 4 displays the recognition accuracy of the three SVM classifiers trained with the deep features obtained from the two layers of each CNN. It is clear from Table 4 that the deep features of either of the two layers of DarkNet-19 attained the highest performance with an accuracy range of 95.6% to 96.4% using the three SVMs. This performance is followed by the deep features of either of the two layers of MobileNet, which achieved an accuracy in the range of 91.6% to 94.0%, while the deep features of either of the two layers of ResNet-18 attained an accuracy that varies between 89.7% and 93.4%. Note that for the cubic SVM classifier, the deep features of layer 1 achieved greater accuracy compared to the deep features of layer 2 for the three CNNs. However, for linear and quadratic SVM classifiers, layer 1 has comparable accuracies, except for the linear SVM classifier fed with deep features of MobileNet and ResNet-18.

### 5.2. Results of the Second Scenario

**Figure 3:**
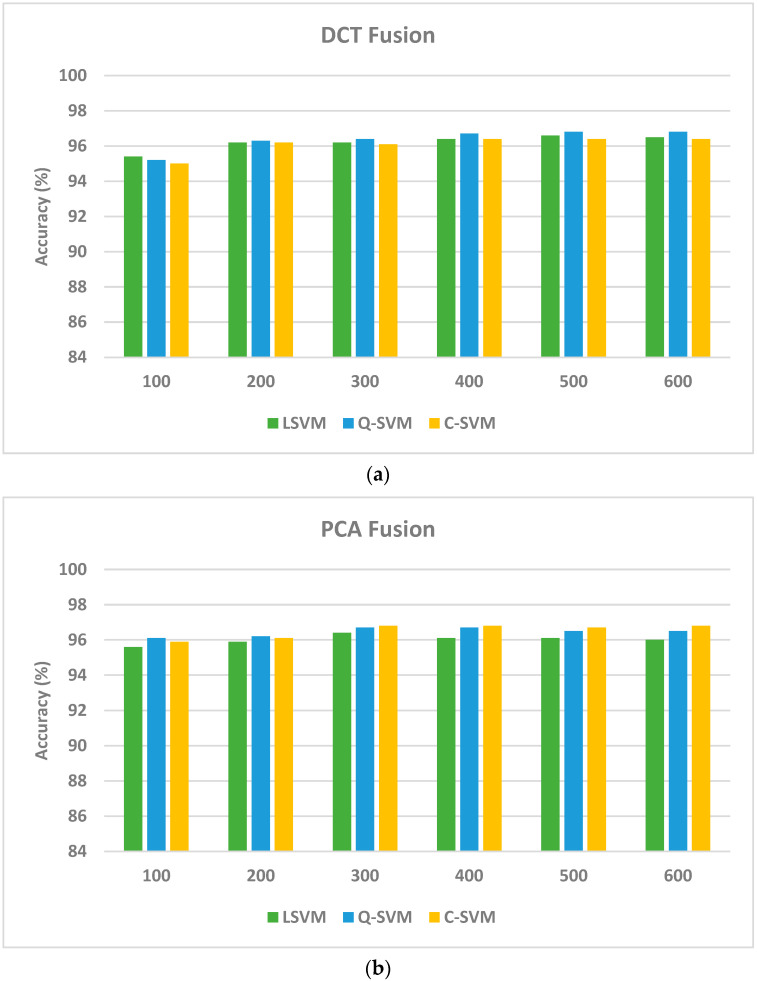
Variation in the recognition accuracy with the number of fused features using both fusion methods: (**a**) DCT and (**b**) PCA.

The goal of this scenario is to verify that the use of deep features of multiple CNNs of different constructions could enhance performance. This section compares the results of the two fusion algorithms that merge layer 1 features and shows that feature fusion could enhance the recognition accuracy of paddy disease using layer 1. An ablation study is accomplished and shown in Figure 3 to demonstrate the variation in accuracy with the number of fused features using both fusion methods (DCT and PCA) for deep layer 1 features. As can be seen in Figure 3, for the DCT method, the recognition accuracy increases until it reaches 96.6%, 96.8%, and 96.4% using the linear, quadratic, and cubic SVM classifiers fed 500 DCT features. Similarly, when using the PCA algorithm to combine deep layer 1 features of the three CNNs, the recognition accuracy improves to 96.4%, 96.7%, and 96.8% by means of the linear, quadratic, and cubic SVM classifiers trained with 300 principal components. These accuracies attained utilizing the two fusion methods are greater than that attained in the first scenario (as indicated in Table 5), which verifies that merging spatial–spectral–temporal features of the three CNNs is capable of enhancing the recognition performance.

Note that the accuracies have improved significantly compared to layer 1 features of MobileNet and ResNet-18 CNNs using the two feature transformation approaches. On the other hand, the accuracies have slightly improved after the fusion step using both feature transformation methods in the case of DarkNet-19. However, the slight improvement in accuracy is accompanied by a great reduction in the number of features employed to train the SVM classifiers, which lowers the complexity of the training models. This is obvious as the accuracies attained after the fusion step using DCT and PCA are (96.6%, 96.4%), (96.8%, 96.7%), and (96.4%, 96.8%) using linear, quadratic, and cubic SVM classifiers. These accuracies are accomplished using only 500 and 300 features by means of DCT and PCA methods which are lower than the 660 features obtained from layer 1 of DarkNet-19 CNN that achieved an accuracy of 96.0%, 96.4%, and 96.3% using the linear, quadratic, and cubic SVM classifiers.

**Figure 4:**
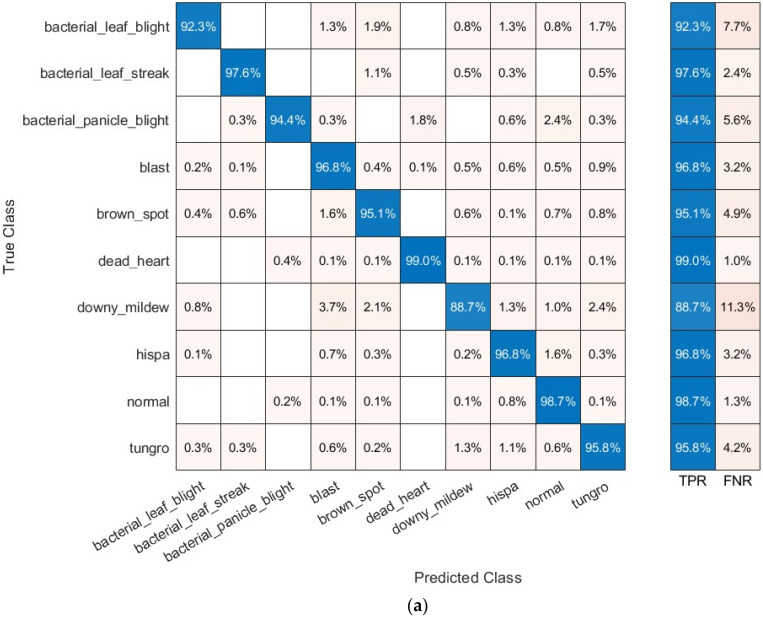

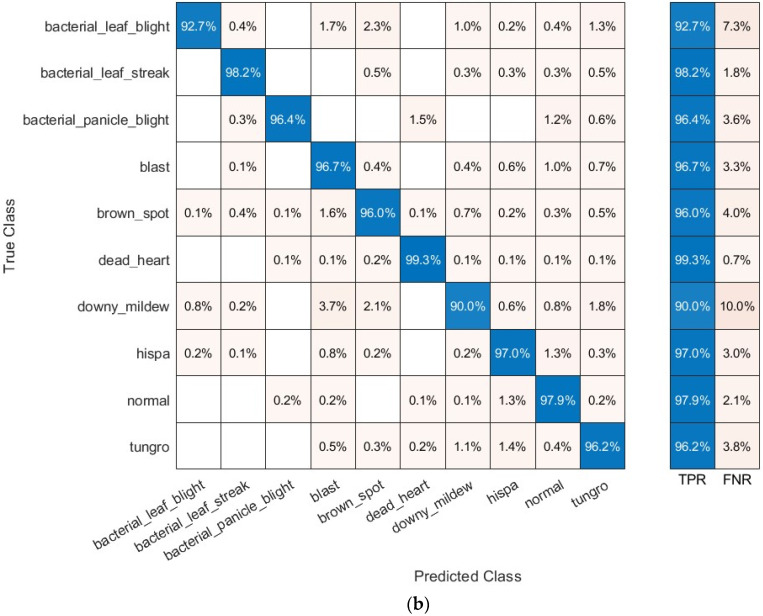
Confusion matrices attained using the quadratic SVM classifier fed with deep features of fused layer 1 using the two fusion methods: (**a**) 500 DCT features and (**b**) 300 PCA features.

The confusion matrices after both fusion algorithms are calculated for the quadratic SVM classifier fed with 500 DCT and 300 PCA coefficients, which achieved the highest accuracy. These confusion matrices are plotted in Figure 4 that specifies the recognition accuracy of each paddy disease. The figure shows that the quadratic SVM classifier has recognized bacterial leaf blight with an accuracy of (92.3% and 92.7%) bacterial leaf streak with an accuracy of (97.6% and 98.2%), bacterial panicle blight with an accuracy of (94.4% and 96.4%), blast with an accuracy of (96.8% and 96.7%), brown spot with an accuracy of (95.1% and 96.0%), dead heart with an accuracy of (99.0% and 99.3%), downy mildew with an accuracy of (88.7% and 90.0%), hispa with an accuracy of (96.8% and 97.0%), tungro with an accuracy of (95.8% and 96.2%), and normal rice leaves with an accuracy of (98.7% and 97.9%) using DCT and PCA features.

### 5.3. Results of the Third Scenario

The third scenario aims to show that combining the two layers of features of the three CNNs and selecting among these features is capable of boosting recognition accuracy. To achieve this goal, the PCA and DCT features are merged independently with layer 2 fused features of the three CNNs; then, the mRMR feature selection approach is applied to choose from these features, thus reducing the feature space dimensionality and lowering the complexity of the recognition process. Table 5 shows the results of the ablation study conducted when changing the number of selected features with the recognition accuracy. Table 5 demonstrates that for PCA Layer 1 Fused Features + Layer 2 Features, the accuracy increases until it accomplishes 97.1%, 97.2%, and 97.2% using linear, quadratic, and cubic SVM classifiers, respectively, with only 300 features. This accuracy is lower than using each fused feature set of each layer separately. Likewise, for DCT Layer 1 Fused Features + Layer 2 Features, the accuracy has improved to 97.3%, 97.5%, and 97.5% with 250 and 300 DCT features using linear, quadratic, and cubic SVM classifiers. These results are higher than those obtained in Table 4 and Figure 4. This improvement in accuracy verifies that feature selection can successfully reduce the number of features and boost recognition accuracy. Furthermore, these results prove that the combination of features from multiple deep layers of distinct CNN structures is capable of enhancing performance.

**Figure 5:**
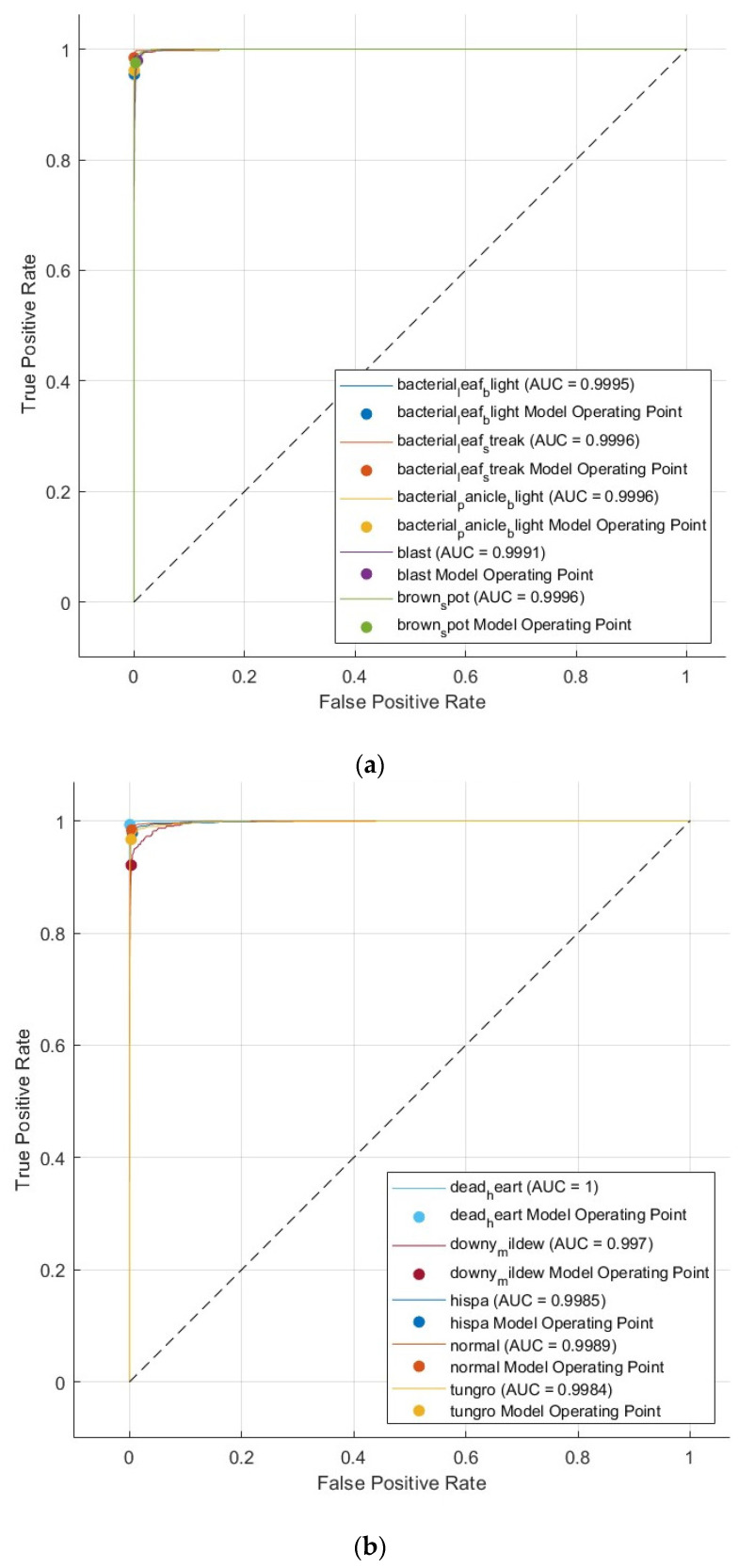
ROC curves for the quadratic SVM classifier trained with the 300 selected deep features that are fused using the DCT method. (**a**) ROC when the positive class is bacterial leaf blight, bacterial leaf streak, bacterial panicle blight, blast, or brown spot class. (**b**) ROC when the positive class is dead heart, downy mildew, hispa, tungro, or normal class.

Other performance measures are also calculated to evaluate the effectiveness of the suggested pipeline with a five-fold cross-validation and hold-out test set. These metrics involve sensitivity, precision, F1-score, specificity, and MCC for the selected features of DCT and PCA. Table 6 reveals these performance measures and indicates that sensitivity ranges from 0.9564 to 0.9707, precision varies between 0.9634 and 0.9742, specificity fluctuates between 0.9959 and 0.9971, F1-score alters between 0.9598 and 0.9722, and MCC changes within the range of 0.9557 to 0.9694 for five-fold cross-validation. Furthermore, the table indicates that when using a hold-out test set, the accuracy fluctuates between 0.9699 and 0.9747, sensitivity ranges from 0.9644 to 0.9724, precision varies between 0.9681 and 0.9750, specificity fluctuates between 0.9966 and 0.9971, F1-score alters between 0.9662 and 0.9736, and MCC changes within the range of 0.9632 to 0.9707. These results show that the proposed pipeline is reliable. Furthermore, the receiving operating characteristic curve (ROC) is also plotted and displayed in Figure 5, and the area under ROC (AUC) is calculated for the quadratic SVM classifier trained with the 300 selected deep features that are fused using the DCT method, as they achieved the highest performance in Table 5.

## 6. Discussion

This research study introduces a pipeline for the automatic recognition of rice paddy diseases. The majority of models used in recent studies operated on databases with few pictures and diseases. However, the suggested pipeline employs a large database with a variety of paddy diseases and a large number of paddy photos. Furthermore, the suggested pipeline deployed three lightweight CNNs of different architectures, fusing the benefits of each composition, contrary to the approaches used in the existing literature, which are based on single CNN models that use a lot of deep layers and a lot of parameters. In contrast to recent research, the suggested pipeline gathers features from two different CNN layers for each. To identify paddy diseases, the suggested pipeline pertains DTCWT to the initial layer’s deep features in order to acquire spectral–temporal information, as opposed to focusing just on spatial knowledge. Additionally, DTCWT is utilized to reduce the size of deep features retrieved from layer 1 and to simplify classification. The suggested pipeline incorporates the deep features of all three lightweight CNNs, as opposed to using the deep features from every CNN separately to carry out recognition. The three CNNs’ layer 1 deep features are merged using PCA and DCT transformation techniques, which further decrease the length of the layer 1 features. After performing a PCA or DCT, it concatenates those integrated spectral–temporal deep features together with the spatial deep features of the second layer. It then provides a feature selection technique to minimize the feature vector dimension and choose only those features that have a significant impact on the recognition process, thereby further lowering recognition complexity.

Three scenarios were used to complete the recognition process. In the first context, the three SVMs are fed with deep features that are obtained from layer 2 and layer 1 after applying DTCWT. Contrarily, in scenario 2, the three SVMs are fed with the combined attributes generated by the two fusion algorithms (PCA and DCT). The three SVMs are then trained using features chosen using the mRMR feature selection approach in scenario 3. The goal of the first scenario is to examine and contrast the performance of the SVM classifier developed with deep features from two different layers of each CNN in terms of recognition. The results of this scenario show that the cubic SVM classifiers trained with layer 1 features are superior to those obtained using layer 2 deep features. These results verify that employing spatial–spectral–temporal features is usually capable of enhancing performance compared to spatial data only. The second scenario aimed to demonstrate how using deep features from various CNNs with different constructions may improve performance. The results of this scenario were better than the first scenario, which proves that merging features from multiple layers improves performance. The purpose of the third scenario was to compare and analyze the SVM classifier’s recognition performance after training it with reduced deep features from two different deep layers selected with the mRMR feature selection approach. The results of this scenario demonstrated that merging features from multiple layers of distinct CNNs can enhance recognition. Furthermore, mRMR feature selection could select a reduced set of features that impact recognition performance. It is worth noting that due to its quick pace and small number of floating-point computation operations, Darknet-53 surpasses other cutting-edge DL techniques, including DenseNet-201 and ResNet-50. Also, Darknet-53 has small-sized (3 × 3 and 1 × 1) subsequent convolutional filters that aid in the identification of objects or structures of different sizes and can accurately discriminate among different patterns [65].

### 6.1. Comparative Evaluation

The efficacy of the suggested pipeline was contrasted with DL frameworks and baseline models that other researchers had previously looked into based on the Paddy Doctor dataset. Note that in the proposed pipeline, there are only nine diseases detected as well as a normal class. In other words, there are 10 classes, including 9 paddy diseases and 1 normal category. Table 7 compares the experimental outcomes of various DL structures with the performance of the suggested pipeline. Table 7 provides evidence that the suggested pipeline is superior to earlier models at identifying the paddy disease. This is because the majority of former models were built on a single DL model, and many of them are complex with huge deep layers and parameters. All of them relied on obtaining deep features from one single deep layer. To select the most important features, these techniques lacked any feature selection approaches. The suggested pipeline, on the other hand, merged deep features of two distinct layers of various lightweight CNNs and employed feature selection, thus improving performance. It also employed spatial as well as spectral–temporal knowledge to achieve recognition. 

### 6.2. Shortcomings and Upcoming Directions

The limitations of this research are primarily focusing on conditions that affect paddy plants rather than all plant illnesses. The topic might be expanded in the upcoming experiments to cover additional plant/crop diseases. Furthermore, the study examined nine paddy diseases as well as one normal condition. Future work will consider more rice diseases. In addition, this study did not employ segmentation and object detection DL models. Forthcoming work will employ segmentation and object detection DL models to examine if they could improve performance. One of the areas that this study did not cover was real-time assessment and implementation, so subsequent research needs to emphasize these areas. The suggestion for the use of chemicals and pesticides depending on the recognized diseases is another worthwhile investigation area that was not addressed in this study. Future research will concentrate on this interesting subject. Additionally, employing image enhancement techniques to make the DL models’ learning process easier will help them distinguish among various ailments of crops. Also, additional elements might be included when collaborating with distinct cultures, such as gathering pictures of multiple species of every crop grown across several nations. The DL models are then trained on these many species to improve their generalization capacity to categorize crops of various species that have been gathered from various cultural contexts. 

## 7. Conclusions

This study proposed a reliable and efficient DL-based pipeline for the automatic recognition of several paddy diseases with close appearance. The findings of the suggested pipeline demonstrated that the two feature transformation methods (PCA and DCT) that were employed separately to fuse layer 1 features of the three CNNs significantly increased accuracies in comparison to layer 1 features of MobileNet, ResNet-18, and DarkNet-19 CNNs. Additionally, the feature selection of the suggested pipeline notably decreased the amount of features utilized in developing the SVM classifiers coinciding with a slight improvement in accuracy, reducing the training models’ complexity. Furthermore, the integration of deep features from the two separate deep levels of the CNNs having different compositions positively impacted identification accuracy. Also, combining the spatial–spectral–temporal attributes of the three CNNs successfully improved recognition performance. In comparison to other prior studies and baseline models for paddy diseases, the results of the proposed pipeline outperformed both models. The outcomes of the suggested pipeline support its efficacy for intelligent agricultural purposes due to its capability to accurately recognize and discriminate paddy illnesses.

## Figures and Tables

**Table 1:** Augmentation techniques used and their range values.

Augmentation Technique	Range
Flip horizontally and vertically	−25 to 25
Scaling	1 to 2
Shearing vertically	−30 to 30
Rotation horizontally and vertically	25 to 25

**Table 2:** Length of feature vectors acquired from the two layers of each CNN.

Model	Layer 1	Layer 1 (After DTCWT)	Layer 2
**ResNet-18**	512	256	10
**DarkNet-19**	660	330	10
**MobileNet**	1280	640	10

**Table 3:** Images distribution along disease categories of the Paddy Doctor dataset.

Paddy Disease Category	Sum of Photos
Blast	1738
Tungro	1088
Dead heart	1442
Bacterial leaf blight	479
Bacterial panicle blight	337
Bacterial leaf streak	380
Hispa	1594
Brown spot	965
Downy mildew	620
Normal	1764

**Table 4:** Accuracy (%) of the classifiers trained with the deep features of the two layers of each CNN.

	ResNet-18		MobileNet		DarkNet-19	
	Layer 1	Layer 2	Layer 1	Layer 2	Layer 1	Layer 2
**LSVM**	89.7	92.5	91.6	93.4	96.0	95.9
**Q-SVM**	92.6	93	93.1	93.4	96.4	96.3
**C-SVM**	93.4	92.7	94.0	92.9	96.3	95.6

**Table 5:** Accuracy (%) of the classifiers trained with the deep features of the two layers of the CNNs.

**PCA Layer 1 Fused Features + Layer 2 Features**						
**Number of Features**						
	**50**	**100**	**150**	**200**	**250**	**300**
**LSVM**	57.4	58.4	81.1	96.4	97.0	97.1
**Q-SVM**	78.1	84.2	91.2	96.7	97.2	97.2
**C-SVM**	78.6	85.3	93.3	96.9	97.4	97.2
**DCT Layer 1 Fused Features + Layer 2 Features**						
**Number of Features**						
	**50**	**100**	**150**	**200**	**250**	**300**
**LSVM**	96.7	97.2	97.2	97.3	97.2	97.3
**Q-SVM**	97.0	97.4	97.5	97.3	97.5	97.5
**C-SVM**	97.0	97.4	97.3	97.5	97.5	97.5

**Table 6:** Performance measures of the classifiers trained with the 300 selected DCT and PCA features of the two layers of the three CNNs.

**PCA Layer 1 Fused Features + Layer 2 Features**						
	**Accuracy**	**Sensitivity**	**Specificity**	**Precision**	**F1-Score**	**MCC**
**Five-Fold Cross-Validation**						
**LSVM**	0.9700	0.9564	0.9959	0.9634	0.9598	0.9557
**Q-SVM**	0.9720	0.9682	0.9968	0.9720	0.9700	0.96692
**C-SVM**	0.9740	0.9687	0.9970	0.9738	0.9712	0.9682
**Hold-Out Test Set**						
**LSVM**	0.9702	0.9661	0.9966	0.9688	0.9674	0.9641
**Q-SVM**	0.9747	0.9721	0.9971	0.9750	0.9734	0.9706
**C-SVM**	0.9747	0.9724	0.9971	0.9750	0.9736	0.9707
**DCT Layer 1 Fused Features + Layer 2 Features**						
	**Accuracy**	**Sensitivity**	**Specificity**	**Precision**	**F1-Score**	**MCC**
**Five-Fold Cross-Validation**						
**LSVM**	0.9730	0.9672	0.9970	0.9721	0.9656	0.9667
**Q-SVM**	0.9750	0.9697	0.9971	0.9742	0.9719	0.9691
**C-SVM**	0.9750	0.9707	0.9971	0.9739	0.9722	0.9694
**Hold-Out Test Set**						
**LSVM**	0.9702	0.9662	0.9966	0.9683	0.9671	0.9638
**Q-SVM**	0.9699	0.9644	0.9966	0.9681	0.9662	0.9628
**C-SVM**	0.9699	0.9657	0.9966	0.9676	0.9666	0.9632

**Table 7:** Comparison of numerical findings with cutting-edge DL models and previous studies for paddy infection recognition using the Paddy Doctor database.

Reference	# Paddy Diseases	Model	Accuracy	F1 Score	Sensitivity	Precision
**[36]**	6	RepVGG	0.9706	0.9709	0.9708	0.9713
**[66]**	10	Custom CNN	0.8888	-	-	-
**[67]**	10	FormerLeaf: a customized vision transformer model	0.9502	0.9616	0.9725	0.9210
**[68]**	10	Swin Transformer	0.9434	0.9343	0.9430	0.9252
**[69]**	10	Convolutional Swin	0.9565	0.9536	0.9615	0.9536
**[70]**	10	Xception	0.9251	0.9155	0.9180	0.9130
**[71]**	10	MobileNet	0.8987	0.9197	0.9348	0.9051
**[72]**	10	ResNet-50	0.9113	0.8933	0.9082	0.8905
**Proposed** **RiPa-Net** **Five-fold cross-validation**	10	MobileNet + ResNet-18 + DarkNet + DCT	0.9750	0.9722	0.9707	0.9739
**Proposed** **RiPa-Net** **Hold-out**		MobileNet + ResNet-18 + DarkNet + PCA	0.9740	0.9666	0.9625	0.9680
		MobileNet + ResNet-18 + DarkNet + DCT + CSVM	0.9699	0.9666	0.9657	0.9676
		MobileNet + ResNet-18 + DarkNet + PCA + CSVM	0.9747	0.9736	0.9724	0.9750

## Data Availability

The dataset can be accessed via the link provided below: https://www.kaggle.com/competitions/paddy-disease-classification (accessed on 10 April 2023). Codes are available at GitHub using the following link: https://github.com/omneya83/Paddy-Disease-Codes.git.

## References

[1] Temniranrat P., Kiratiratanapruk K., Kitvimonrat A., Sinthupinyo W., Patarapuwadol S. (2021). A System for Automatic Rice Disease Detection from Rice Paddy Images Serviced via a Chatbot. Comput. Electron. Agric..

[2] Rehman A.U., Farooq M., Rashid A., Nadeem F., Stuerz S., Asch F., Bell R.W., Siddique K.H. (2018). Boron Nutrition of Rice in Different Production Systems. A Review. Agron. Sustain. Dev..

[3] Shrivastava V.K., Pradhan M.K., Minz S., Thakur M.P. (2019). Rice Plant Disease Classification Using Transfer Learning of Deep Convolution Neural Network. Int. Arch. Photogramm. Remote Sens. Spat. Inf. Sci..

[4] Zhang X., Han L., Dong Y., Shi Y., Huang W., Han L., González-Moreno P., Ma H., Ye H., Sobeih T. (2019). A Deep Learning-Based Approach for Automated Yellow Rust Disease Detection from High-Resolution Hyperspectral UAV Images. Remote Sens..

[5] Tholkapiyan M., Aruna Devi B., Bhatt D., Saravana Kumar E., Kirubakaran S., Kumar R. (2023). Performance Analysis of Rice Plant Diseases Identification and Classification Methodology. Wirel. Pers. Commun..

[6] Attallah O. (2023). GabROP: Gabor Wavelets-Based CAD for Retinopathy of Prematurity Diagnosis via Convolutional Neural Networks. Diagnostics.

[7] Attallah O. (2022). A Computer-Aided Diagnostic Framework for Coronavirus Diagnosis Using Texture-Based Radiomics Images. Digit. Health.

[8] Attallah O. (2023). RADIC: A Tool for Diagnosing COVID-19 from Chest CT and X-Ray Scans Using Deep Learning and Quad-Radiomics. Chemom. Intell. Lab. Syst..

[9] Aurna N.F., Yousuf M.A., Taher K.A., Azad A.K.M., Moni M.A. (2022). A Classification of MRI Brain Tumor Based on Two Stage Feature Level Ensemble of Deep CNN Models. Comput. Biol. Med..

[10] Attallah O. Deep Learning-Based CAD System for COVID-19 Diagnosis via Spectral-Temporal Images. Proceedings of the 2022 the 12th International Conference on Information Communication and Management.

[11] Attallah O. (2021). MB-AI-His: Histopathological Diagnosis of Pediatric Medulloblastoma and Its Subtypes via AI. Diagnostics.

[12] Attallah O. (2022). An Intelligent ECG-Based Tool for Diagnosing COVID-19 via Ensemble Deep Learning Techniques. Biosensors.

[13] Aggarwal S., Chugh N., Balyan A. (2023). Identification of ADHD Disorder in Children Using EEG Based on Visual Attention Task by Ensemble Deep Learning. Proceedings of the International Conference on Data Science and Applications: ICDSA 2022.

[14] Attallah O. (2023). Cervical Cancer Diagnosis Based on Multi-Domain Features Using Deep Learning Enhanced by Handcrafted Descriptors. Appl. Sci..

[15] Gong W., Chen H., Zhang Z., Zhang M., Wang R., Guan C., Wang Q. (2019). A Novel Deep Learning Method for Intelligent Fault Diagnosis of Rotating Machinery Based on Improved CNN-SVM and Multichannel Data Fusion. Sensors.

[16] Mandal S., Santhi B., Sridhar S., Vinolia K., Swaminathan P. (2017). Nuclear Power Plant Thermocouple Sensor-Fault Detection and Classification Using Deep Learning and Generalized Likelihood Ratio Test. IEEE Trans. Nucl. Sci..

[17] Attalah O. (2023). Multitask Deep Learning-Based Pipeline for Gas Leakage Detection via E-Nose and Thermal Imaging Multimodal Fusion. Chemosensors.

[18] Rahate A., Mandaokar S., Chandel P., Walambe R., Ramanna S., Kotecha K. (2022). Employing Multimodal Co-Learning to Evaluate the Robustness of Sensor Fusion for Industry 5.0 Tasks. Soft Comput..

[19] Attallah O. (2023). Tomato Leaf Disease Classification via Compact Convolutional Neural Networks with Transfer Learning and Feature Selection. Horticulturae.

[20] Javidan S.M., Banakar A., Vakilian K.A., Ampatzidis Y. (2023). Diagnosis of Grape Leaf Diseases Using Automatic K-Means Clustering and Machine Learning. Smart Agric. Technol..

[21] Munjal D., Singh L., Pandey M., Lakra S. (2023). A Systematic Review on the Detection and Classification of Plant Diseases Using Machine Learning. Int. J. Softw. Innov..

[22] Thakur P.S., Sheorey T., Ojha A. (2023). VGG-ICNN: A Lightweight CNN Model for Crop Disease Identification. Multimed. Tools Appl..

[23] Tabbakh A., Barpanda S.S. (2023). A Deep Features Extraction Model Based on the Transfer Learning Model and Vision Transformer “TLMViT” for Plant Disease Classification. IEEE Access.

[24] Sethy P.K., Barpanda N.K., Rath A.K., Behera S.K. (2020). Deep Feature Based Rice Leaf Disease Identification Using Support Vector Machine. Comput. Electron. Agric..

[25] Adeel A., Khan M.A., Akram T., Sharif A., Yasmin M., Saba T., Javed K. (2022). Entropy-Controlled Deep Features Selection Framework for Grape Leaf Diseases Recognition. Expert Syst..

[26] Peng Y., Zhao S., Liu J. (2021). Fused-Deep-Features Based Grape Leaf Disease Diagnosis. Agronomy.

[27] Koklu M., Unlersen M.F., Ozkan I.A., Aslan M.F., Sabanci K. (2022). A CNN-SVM Study Based on Selected Deep Features for Grapevine Leaves Classification. Measurement.

[28] Farooqui N.A., Mishra A.K., Mehra R. (2022). Concatenated Deep Features with Modified LSTM for Enhanced Crop Disease Classification. Int. J. Intell. Robot. Appl..

[29] Attallah O. (2023). CerCan·Net: Cervical Cancer Classification Model via Multi-Layer Feature Ensembles of Lightweight CNNs and Transfer Learning. Expert Syst. Appl..

[30] Attallah O. (2023). MonDiaL-CAD: Monkeypox Diagnosis via Selected Hybrid CNNs Unified with Feature Selection and Ensemble Learning. Digit. Health.

[31] Attallah O. (2022). ECG-BiCoNet: An ECG-Based Pipeline for COVID-19 Diagnosis Using Bi-Layers of Deep Features Integration. Comput. Biol. Med..

[32] Attallah O., Zaghlool S. (2022). AI-Based Pipeline for Classifying Pediatric Medulloblastoma Using Histopathological and Textural Images. Life.

[33] Aggarwal M., Khullar V., Goyal N. Contemporary and Futuristic Intelligent Technologies for Rice Leaf Disease Detection. Proceedings of the 2022 10th International Conference on Reliability, Infocom Technologies and Optimization (Trends and Future Directions) (ICRITO).

[34] Maheswaran S., Sathesh S., Rithika P., Shafiq I.M., Nandita S., Gomathi R.D. (2022). Detection and Classification of Paddy Leaf Diseases Using Deep Learning (CNN). Computer, Communication, and Signal Processing, Proceedings of the 6th IFIP TC 5 International Conference, ICCCSP 2022, Chennai, India, 24–25 February 2022.

[35] Upadhyay S.K., Kumar A. (2021). Early-Stage Brown Spot Disease Recognition in Paddy Using Image Processing and Deep Learning Techniques. Trait. Signal.

[36] Ni H., Shi Z., Karungaru S., Lv S., Li X., Wang X., Zhang J. (2023). Classification of Typical Pests and Diseases of Rice Based on the ECA Attention Mechanism. Agriculture.

[37] Haridasan A., Thomas J., Raj E.D. (2023). Deep Learning System for Paddy Plant Disease Detection and Classification. Environ. Monit. Assess..

[38] Akyol K. (2022). Handling Hypercolumn Deep Features in Machine Learning for Rice Leaf Disease Classification. Multimed. Tools Appl..

[39] Yakkundimath R., Saunshi G., Anami B., Palaiah S. (2022). Classification of Rice Diseases Using Convolutional Neural Network Models. J. Inst. Eng. (India) Ser. B.

[40] Lamba S., Baliyan A., Kukreja V. (2023). A Novel GCL Hybrid Classification Model for Paddy Diseases. Int. J. Inf. Technol..

[41] Sethy P.K., Behera S.K., Kannan N., Narayanan S., Pandey C. (2021). Smart Paddy Field Monitoring System Using Deep Learning and IoT. Concurr. Eng..

[42] Saleem M.A., Aamir M., Ibrahim R., Senan N., Alyas T. (2022). An Optimized Convolution Neural Network Architecture for Paddy Disease Classification. Comput. Mater. Contin..

[43] Attallah O. (2021). CoMB-Deep: Composite Deep Learning-Based Pipeline for Classifying Childhood Medulloblastoma and Its Classes. Front. Neuroinform..

[44] Pramanik R., Biswas M., Sen S., de Souza Júnior L.A., Papa J.P., Sarkar R. (2022). A Fuzzy Distance-Based Ensemble of Deep Models for Cervical Cancer Detection. Comput. Methods Programs Biomed..

[45] Mallat S.G. (1989). A Theory for Multiresolution Signal Decomposition: The Wavelet Representation. IEEE Trans. Pattern Anal. Mach. Intell..

[46] Mallat S. (1999). A Wavelet Tour of Signal Processing.

[47] Cohen A., Daubechies I., Jawerth B., Vial P. (1993). Multiresolution Analysis, Wavelets and Fast Algorithms on an Interval. C. R. Acad. Sci. Sér. 1 Math..

[48] Cohen A., Daubechies I., Feauveau J.-C. (1992). Biorthogonal Bases of Compactly Supported Wavelets. Commun. Pure Appl. Math..

[49] Wold S., Esbensen K., Geladi P. (1987). Principal Component Analysis. Chemom. Intell. Lab. Syst..

[50] Dunteman G.H. (1989). Principal Components Analysis.

[51] Ahmed N., Natarajan T., Rao K.R. (1974). Discrete Cosine Transform. IEEE Trans. Comput..

[52] Strang G. (1999). The Discrete Cosine Transform. SIAM Rev..

[53] Narasimha M., Peterson A. (1978). On the Computation of the Discrete Cosine Transform. IEEE Trans. Commun..

[54] Ochoa-Dominguez H., Rao K.R. (2019). Discrete Cosine Transform.

[55] Attallah O., Samir A. (2022). A Wavelet-Based Deep Learning Pipeline for Efficient COVID-19 Diagnosis via CT Slices. Appl. Soft Comput..

[56] Jogin M., Madhulika M.S., Divya G.D., Meghana R.K., Apoorva S. Feature Extraction Using Convolution Neural Networks (CNN) and Deep Learning. Proceedings of the 2018 3rd IEEE International Conference on Recent Trends in Electronics, Information & Communication Technology (RTEICT).

[57] Cai J., Luo J., Wang S., Yang S. (2018). Feature Selection in Machine Learning: A New Perspective. Neurocomputing.

[58] Radovic M., Ghalwash M., Filipovic N., Obradovic Z. (2017). Minimum Redundancy Maximum Relevance Feature Selection Approach for Temporal Gene Expression Data. BMC Bioinform..

[59] Thai L.H., Hai T.S., Thuy N.T. (2012). Image Classification Using Support Vector Machine and Artificial Neural Network. Int. J. Inf. Technol. Comput. Sci..

[60] Attallah O. (2022). A Deep Learning-Based Diagnostic Tool for Identifying Various Diseases via Facial Images. Digit. Health.

[61] Attallah O. (2021). DIAROP: Automated Deep Learning-Based Diagnostic Tool for Retinopathy of Prematurity. Diagnostics.

[62] Murugan D. (2022). Paddy Doctor: A Visual Image Dataset for Paddy Disease Classification. arXiv.

[63] Keskar N.S., Mudigere D., Nocedal J., Smelyanskiy M., Tang P.T.P. (2016). On Large-Batch Training for Deep Learning: Generalization Gap and Sharp Minima. arXiv.

[64] Li M., Zhang T., Chen Y., Smola A.J. Efficient Mini-Batch Training for Stochastic Optimization. Proceedings of the 20th ACM SIGKDD International Conference on Knowledge Discovery and Data Mining.

[65] Ullah A., Muhammad K., Ding W., Palade V., Haq I.U., Baik S.W. (2021). Efficient Activity Recognition Using Lightweight CNN and DS-GRU Network for Surveillance Applications. Appl. Soft Comput..

[66] Nayem Z.H., Jahan I., Rakib A.A., Mia S. Detection and Identification of Rice Pests Using Memory Efficient Convolutional Neural Network. Proceedings of the 2023 International Conference on Computer, Electrical & Communication Engineering (ICCECE).

[67] Thai H.-T., Le K.-H., Nguyen N.L.-T. (2023). FormerLeaf: An Efficient Vision Transformer for Cassava Leaf Disease Detection. Comput. Electron. Agric..

[68] Wang F., Rao Y., Luo Q., Jin X., Jiang Z., Zhang W., Li S. (2022). Practical Cucumber Leaf Disease Recognition Using Improved Swin Transformer and Small Sample Size. Comput. Electron. Agric..

[69] Guo Y., Lan Y., Chen X. (2022). CST: Convolutional Swin Transformer for Detecting the Degree and Types of Plant Diseases. Comput. Electron. Agric..

[70] Chollet F. Xception: Deep Learning with Depthwise Separable Convolutions. Proceedings of the IEEE Conference on Computer Vision and Pattern Recognition.

[71] Howard A.G., Zhu M., Chen B., Kalenichenko D., Wang W., Weyand T., Andreetto M., Adam H. (2017). Mobilenets: Efficient Convolutional Neural Networks for Mobile Vision Applications. arXiv.

[72] He K., Zhang X., Ren S., Sun J. Deep Residual Learning for Image Recognition. Proceedings of the IEEE Conference on Computer Vision and Pattern Recognition.

